# Supplementary material for: The Potentiating Effect of Graphene Oxide on the Arylhydrocarbon Receptor (AhR)–Cytochrome P4501A (Cyp1A) System Activated by Benzo(k)fluoranthene (BkF) in Rainbow Trout Cell Line
Source: Nanomaterials (Basel). 2023 Sep 5;13(18):2501. doi: 10.3390/nano13182501 (PMC10534689; doi:10.3390/nano13182501)
Supplement: Supplementary file 1 [file nanomaterials-13-02501-s001.zip › nanomaterials-2564414-supplementary.pdf]

Table S1: Primers for quantitative PCR analysis of gene expression.

Target gene	Primers sequence (5. . .3)	Product size (bp)	Reference
eef-1a	Sense: TGCCCCTGGACACAGAGATT<br>Antisense: CCCACACCACCAGCAACAA	90	[61]
cyp1a	Sense: TCAACTTACCTCTGCTGGAAGC<br>Antisense: GATGAACGGCAGGAAGGA	68	[62]
ahr	Sense: GTGTTCTATGCCTCTCCTACTATC<br>Antisense: GTCATCTGTGTGGATCAGCTCAA	90	[63]

Table S2: Fluorescence quenching: dose dependent attenuation of fluorescence intensity caused by GO.

GO g/ml	Resorufin EROD (M)		Resorufin AB (M)		HFC BFCOD (M)		5′-CF (M)		NR (mg/ml)		ROS (M)	
	5	0.5	1	0.1	40	4	4	0.4	0.3	0.03	100	10
75	16.5	11.00	16.37	17.76	16.5	15.41	10.86	8.03	7.62	9.38	5.67	7.68
37.5	8.68	5.73	8.35	6.92	11.03	6.78	7.56	0.09	0	1.74	1.98	1.05
18.75	3.42	1.41	2.94	1.79	3.39	0.5	1.29	0.4	0	0.20	0.5	0.3
9.4	5.8	1.64	2.87	0	2.66	0.6	3.79	0	0	1.18	0	0.1
4.7	6	0	0.27	0	3.1	0.75	1.62	0	0	0	0	0
2.3	1.63	0	2.87	0	3.72	0.07	0	0	0	0	0	0
1.17	0	0	0	0	0	0	0	0	0	0	0	0
0.6	0	0	0	0	0	0	0	0	0	0	0	0
0.3	0	0	0	0	0	0	0	0	0	0	0	0

For all fluorophores a dose dependent attenuation of fluorescence intensity (%) was determined. Only at the highest concentration of GO suspension tested (75 g/ml) was the degree of quenching between 10% and 20%.The degree of quenching was independent of the fluorophore concentration.

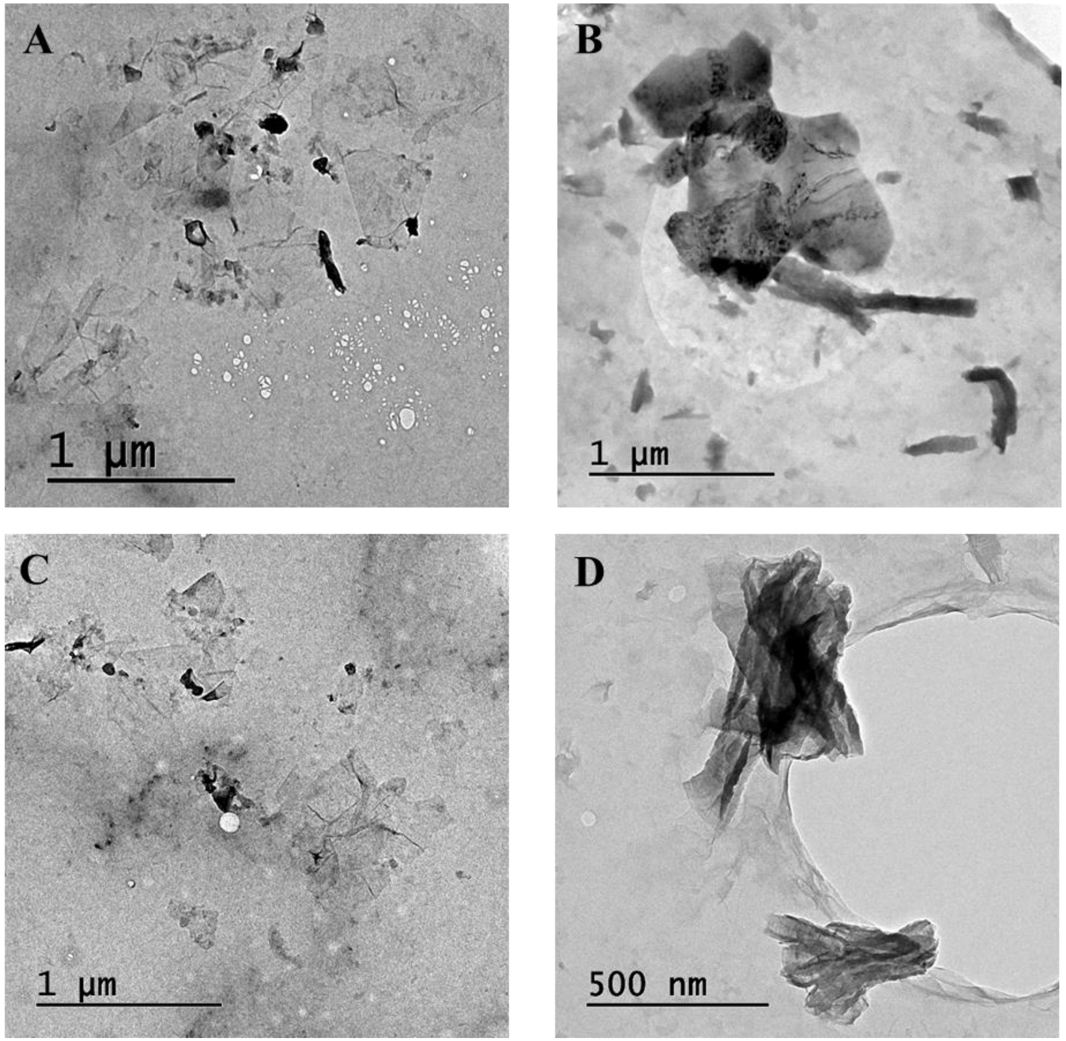

Figure S1. TEM micrographs of GO suspensions in MiliQ water (A, C) and in L-15 media (B, D) confirmed the presence of single or few layer graphene sheets, The images revealed no remarkable differences in size or shape between Milli-Q water (Fig.S1A, C) and L-15 media dispersions (Fig. S1B, D at different exposure times; 0 days (A, B) and 7 days (C, D).

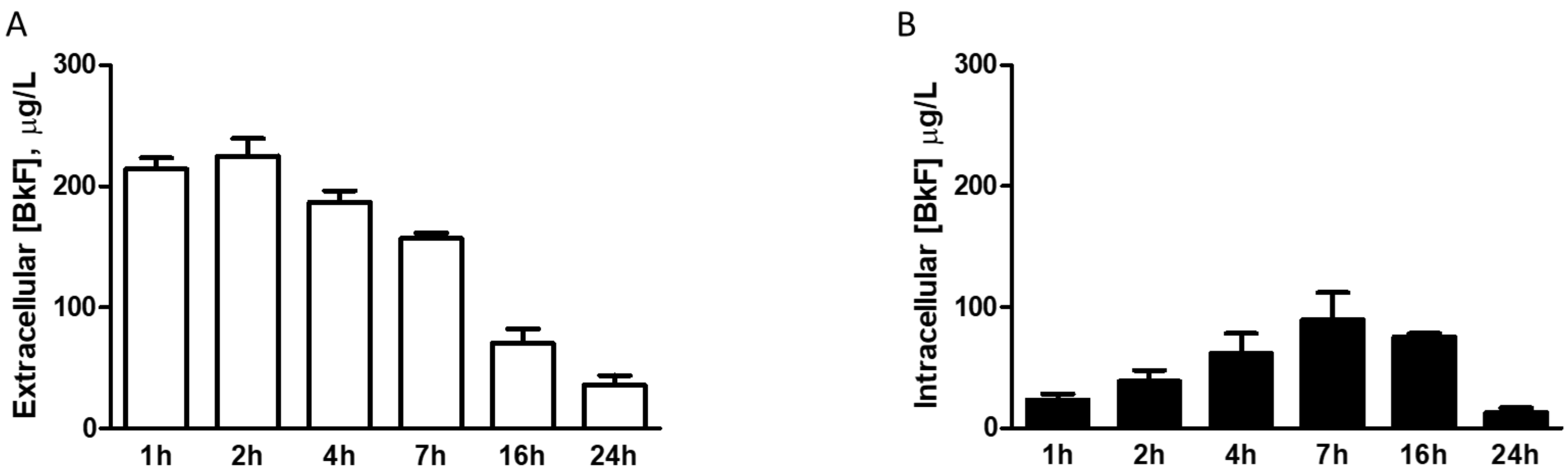

Figure S2. Extracellular (A) and intracellular (B) BkF levels assessed by means of SPE-HPLC-FLD. RTL-W1 cells were exposed to BkF at 1 M (252.31 g/L) for different times (1h to 24h, period corresponding to pre-exposure to BkF). Media and cells were collected and processed to BkF determination.
